# Atypical Haemolytic Uraemic Syndrome Associated with a Hybrid Complement Gene

**DOI:** 10.1371/journal.pmed.0030431

**Published:** 2006-10-31

**Authors:** Julian P Venables, Lisa Strain, Danny Routledge, David Bourn, Helen M Powell, Paul Warwicker, Martha L Diaz-Torres, Anne Sampson, Paul Mead, Michelle Webb, Yves Pirson, Michael S Jackson, Anne Hughes, Katrina M Wood, Judith A Goodship, Timothy H. J Goodship

**Affiliations:** 1 Institute of Human Genetics, University of Newcastle upon Tyne, Newcastle upon Tyne, United Kingdom; 2 Northern Molecular Genetics Service Laboratory, Newcastle upon Tyne Hospitals National Health Service Trust, Newcastle upon Tyne, United Kingdom; 3 Renal Unit, East and North Hertfordshire National Health Service Trust, Stevenage, United Kingdom; 4 Department of Immunology, Newcastle upon Tyne Hospitals National Health Service Trust, Newcastle upon Tyne, United Kingdom; 5 Renal Unit, North Cumbria Acute Hospitals National Health Service Trust, Carlisle, United Kingdom; 6 Renal Unit, East Kent Hospitals National Health Service Trust, Canterbury, United Kingdom; 7 Service de Nephrologie, Cliniques Universitaires Saint-Luc, Brussels, Belgium; 8 Department of Medical Genetics, Queen's University, Belfast, United Kingdom; 9 Department of Histopathology, Newcastle upon Tyne Hospitals National Health Service Trust, Newcastle upon Tyne, United Kingdom; Instituto Mario Negri, Italy

## Abstract

**Background:**

Sequence analysis of the regulators of complement activation (RCA) cluster of genes at chromosome position 1q32 shows evidence of several large genomic duplications**.** These duplications have resulted in a high degree of sequence identity between the gene for factor H *(CFH)* and the genes for the five factor H-related proteins *(CFHL1–5;* aliases *CFHR1–5)*. *CFH* mutations have been described in association with atypical haemolytic uraemic syndrome (aHUS). The majority of the mutations are missense changes that cluster in the C-terminal region and impair the ability of factor H to regulate surface-bound C3b. Some have arisen as a result of gene conversion between *CFH* and *CFHL1*. In this study we tested the hypothesis that nonallelic homologous recombination between low-copy repeats in the RCA cluster could result in the formation of a hybrid *CFH/CFHL1* gene that predisposes to the development of aHUS.

**Methods and Findings:**

In a family with many cases of aHUS that segregate with the RCA cluster we used cDNA analysis, gene sequencing, and Southern blotting to show that affected individuals carry a heterozygous *CFH/CFHL1* hybrid gene in which exons 1–21 are derived from *CFH* and exons 22/23 from *CFHL1*. This hybrid encodes a protein product identical to a functionally significant *CFH* mutant (c.3572C>T, S1191L and c.3590T>C, V1197A) that has been previously described in association with aHUS.

**Conclusions:**

*CFH* mutation screening is recommended in all aHUS patients prior to renal transplantation because of the high risk of disease recurrence post-transplant in those known to have a *CFH* mutation. Because of our finding it will be necessary to implement additional screening strategies that will detect a hybrid *CFH/CFHL1* gene.

## Abstract 2

Tim Goodship and colleagues have identified a heterozygous*CFH/CFHL1* hybrid gene which encodes a protein product identical to one previously described in association with atypical hemolytic uremic syndrome.

## Editors' Summary

**Background.:**

Atypical hemolytic uremic (aHUS) syndrome is a rare, chronic disease that can run in families. People with the condition are prone to developing kidney failure and high blood pressure, and are likely to have a shorter life span than healthy people. Previous work done by a group of researchers in Newcastle-on-Tyne, UK looked at the genetic underpinnings of aHUS in three families suffering from the condition. They found a region of the genome that was linked with the disease in all three families. That region was known to contain a gene for a protein called “factor H,” as well as a number of other genes for proteins that are involved in the same pathway as factor H in controlling an ancient defence system called complement. This system helps antibodies to kill invaders by marking any cell that is not protected by proteins such as factor H. Our own cells would be under constant threat without protective proteins such as factor H. Later studies found simple genetic mutations in people with aHUS, in the genes coding for factor H. However, other work suggested that in some families with aHUS, simple genetic mutations might not be the cause; instead more complicated rearrangements of the genome might occur which would then result in an abnormal factor H that incorporated part of the gene for another protective protein called factor H related protein 1.

**Why Was This Study Done?:**

The researchers knew that it was important to understand the exact genetic mutations linked with aHUS in different families. This was because the exact type of mutation would help them predict whether a kidney transplant is likely to be successful in treating an individual with aHUS who has developed kidney failure. In people with mutations affecting proteins produced by the kidney, a kidney transplant would be likely to work; but in people with mutations affecting factor H, which is produced by the liver, the disease would probably recur after a kidney transplant.

**What Did the Researchers Do and Find?:**

In this study, the researchers went back to one of the three families with aHUS they had previously studied. The researchers had shown before that in this family, the disease was linked with the genome region containing factor H, but no precise mutation in that region had been found. This time, the researchers screened the genome of the family members and looked in particular for a specific rearrangement of the genome that they suspected might be involved. They found that the genomes in this family had been shuffled in the factor H region, resulting in an abnormal version of factor H being produced.

**What Do These Findings Mean?:**

The mutation these researchers identified is likely to result in development of aHUS that does not get better after a kidney transplant, because the abnormal factor H would still be produced in the liver after a transplant had been done. Therefore, the researchers suggest that patients with aHUS be checked for this particular mutation before it is decided whether to go ahead with a transplant.

**Additional Information.:**

Please access these Web sites via the online version of this summary at http://dx.doi.org/10.1371/journal.pmed.0030431.

- US National Institutes of Health Office of Rare Diseases information about atypical hemolytic uremic syndrome
- The Online Mendelian Inheritance in Man (OMIM) contains an entry on hemolytic uremic syndrome. OMIM is a database of human genes and genetic disorders developed by the US National Center for Biotechnology Information
- The US National Kidney and Urologic Diseases has a page about hemolytic uremic syndrome
- The Wikipedia has a page about HUS (note that Wikipedia is a free online encyclopedia that anyone can edit)

## Introduction

Atypical HUS (aHUS) is characterised by the triad of a microangiopathic haemolytic anaemia (Coombs' test negative), thrombocytopenia, and acute renal failure in the absence of a preceding diarrhoeal illness. aHUS can be either sporadic or, if more than one member of a family is affected, familial. In 1998 we established linkage in three families with atypical haemolytic uraemic syndrome (aHUS) to a 26 cM region at chromosome location 1q32 [1]. This area contains a group of genes that play a pivotal role in the regulation of complement activation (the regulators of complement activation [RCA] cluster). In one of these families we found a mutation in the gene encoding the soluble regulator complement factor H [1] and subsequently in another we found a mutation in the gene encoding membrane cofactor protein, a transmembrane regulator [2]. In the third family extensive screening of genes within the RCA cluster failed to reveal an abnormality.

**Figure 1:**
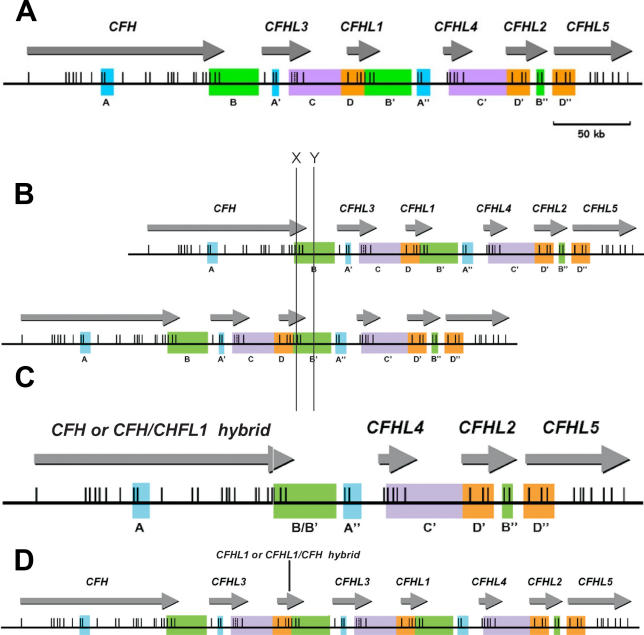
Genomic Structure of the Region of the RCA Cluster Containing the Genes Encoding Factor H and the Five factor H-Related Proteins Genomic duplications including the different exons of the six genes were originally determined by Male et al. [4] and are colour-coded. Exons are indicated as vertical lines. (A) Position of the genes encoding factor H and the factor H-related proteins in a centromeric segment of the RCA cluster at 1q32. (B and C) Nonhomologous recombination occurring at X would result in a hybrid gene consisting of the first 21 exons of *CFH* (encoding SCRs 1–18 of the hybrid gene) and the last 2 exons of *CFHL1* (encoding SCRs 19 and 20 of the hybrid gene). If the recombination occurred at Y this would result in deletion of *CFHL3* and *CFHL1* but *CFH* would remain intact. (D) The recombination event would also potentially lead to a duplication of *CFHL1* and *CFHL3*. (Figure adapted from Figure 1 of [15] with kind permission of Human Mutation C 2006, Wiley Liss Inc., A Wiley Company.)

Complement genes within the RCA cluster are arranged in tandem within two groups [3]. In a centromeric 360 kb segment lie the genes encoding factor H *(CFH)* and five factor H-related proteins—*CFHL1*–*5* (aliases *CFHR1–5*) (Figure 1A). Sequence analysis of this region shows evidence of several large genomic duplications, also known as low copy repeats (LCRs), resulting in a high degree of sequence identity between *CFH* and the genes for the five factor H-related proteins [4,5]. The secreted protein products of these genes are similar in that they consist of repetitive units (~60 amino acids) named short consensus repeats (SCRs) or complement control protein modules. Each SCR is generally encoded by a single exon. CFH consists of 20 SCRs and CFHL1 consists of five. The highest degree of sequence identity is seen between SCRs 18 and 20 of CFH and SCRs 3 and 5 of CFHL1. CFH SCR 18 and CFHL1 SCR 3 consist of 59 amino acids. At the nucleotide level the exons encoding these two SCRs differ by five bases. Three result in an amino acid difference. CFH has tyrosine, valine, and glutamine residues at positions 1,058, 1,060, and 1,076, respectively (encoded by triplets **T**AT, **G**TG, and **C**AA), whilst CFHL1 has histidine, leucine, and glutamic acid residues at positions 157, 159, and 175 (encoded by triplets **C**AT, **C**TG and **G**AA). CFH SCR 19 and CFHL1 SCR 4 consist of 61 amino acids. The exons encoding these two SCRs differ at one nucleotide position, which does not result in a coding change. CFH SCR20 and CFHL1 SCR 5 consist of 67 amino acids. The exons differ at two nucleotide positions, both of which affect the amino acid sequence of the encoded proteins; CFH has serine and valine residues at positions 1,191 and 1,197 (encoded by triplets T**C**G and G**T**T) while CFHL1 has leucine and alanine residues at positions 290 and 296 (encoded by triplets T**T**G and G**C**T). Mutations reported in association with aHUS include c.3572C>T, which results in the change S1191L and c.3590T>C, which results in V1197A, either singly or in combination [6–8]. This raised the possibility that gene conversion of CFH SCR 20 by SCR 5 of CFHL1 is the mutational mechanism in a proportion of cases. c.3226C>G, Q1076E has also been reported [6,9], raising the possibility of gene conversion of CFH SCR 18 by SCR 3 of CFHL1.

We recently reported two aHUS cases in which S1191L and V1197A changes occurred in combination as de novo events [10], thus providing unambiguous evidence that gene conversion is the mutational mechanism involved. LCRs such as those seen in the RCA cluster not only predispose to gene conversion events but are also associated with genomic rearrangements [11]. These rearrangements usually result from nonallelic homologous recombination between LCRs. If nonallelic recombination should occur between the duplicated segments B and B′ shown in Figure 1B, a variety of products are possible (Figure 1C and 1D). If recombination occurred after the terminal exons of *CFH* and *CFHL1* deletion of *CFHL1* and *CFHL3* would result. If recombination occurred within the region containing the three terminal exons (and their flanking introns) of *CFH* and *CFHL1,* then not only would *CFHL3* be deleted, but a hybrid *CFH/CFHL1* gene would be formed. If, for example, the recombination occurred at “X” in Figure 1B, a hybrid gene would result that consisted of the first 21 exons of *CFH* (encoding SCRs 1–18 of the hybrid gene) and the last two exons of *CFHL1* (encoding SCRs 19 and 20 of the hybrid gene) (Figure 1C). The hybrid gene would encode a protein product identical to the aforementioned S1191L/V1197A mutant, a change that we have shown to be functionally significant [10]. If the recombination occurred at Y, *CFHL3* and *CFHL1* would be deleted, but *CFH* would remain intact (Figure 1C). The recombination event would also potentially lead to duplication of *CFHL1* and *CFHL3* (Figure 1D). There is already evidence that nonallelic recombination leading to deletion of *CFHL1* occurs as a common polymorphism in the general population [12], and we here show that this phenomenon also leads to the formation of a hybrid *CFH/CFHL1* gene associated with aHUS. In particular, we have shown the presence of such a hybrid gene in the affected members of the remaining unsolved family from our original linkage study [1].

## Methods

### Clinical Details and Informed Consent

**Figure 2:**
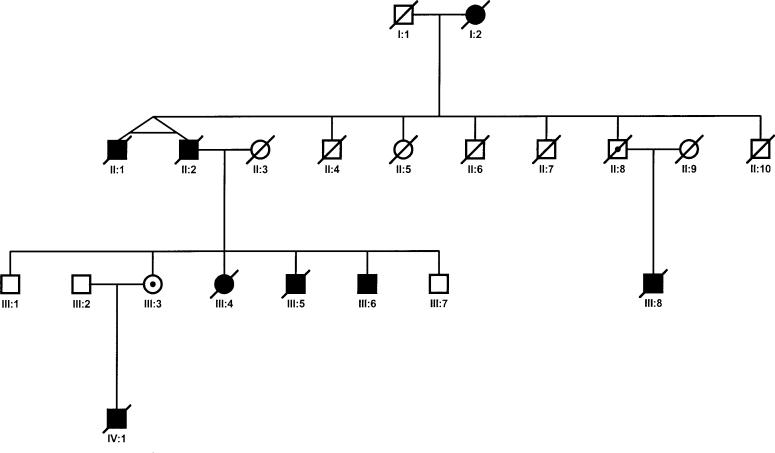
Pedigree of the Family

The family that is the subject of our study was first reported in 1975 [13] and more recently in 1998 [1]. This is the aforementioned one remaining unsolved family from our original linkage study in three aHUS families in 1998 [1]. The current pedigree is shown in Figure 2. Affected member I:2 died at the age of 57 y, 7 d after presenting with pericarditis, heart failure, and hypertension.

II:1 presented with the classical features of HUS at the age of 45 y, following a 9 mo history of hypertension. Serum complement levels were normal. Renal function did not recover and haemodialysis was commenced. Bilateral nephrectomy was undertaken because of ongoing haemolysis and severe hypertension. He subsequently received two renal transplants, one at the age of 46 y and the other at 49 y. Both kidneys were lost to “acute rejection,” the first after 18 mo and the second after 8 mo. In retrospect the clinical and histopathological features were compatible with recurrent HUS. He died of a myocardial infarct at the age of 55 y.

II:2, the twin brother of II:1, presented at the age of 46 y with acute renal failure and hypertension. Investigations were compatible with a diagnosis of HUS. As with his brother, bilateral nephrectomy was necessary to control ongoing haemolysis and hypertension. He subsequently received two transplants, one at the age of 47 y and the other at 60 y. Both kidneys were lost within weeks of transplantation due to recurrent HUS. II:7 and II:10 both died of acute renal failure. II:8 died at the age of 82 y without any evidence of renal disease.

III:3 is currently aged 53 y and is well. Complement profiles have shown a persistently borderline low C3. III:4 presented at the age of 19 y with a grand mal convulsion. She had started an oral contraceptive 6 wk previously. Investigations were consistent with HUS. A complement profile was said to be normal. She died 8 wk after presentation following cardiac arrest. III:5 presented with HUS at the age of 5 y and died 6 d after presentation. III:6 presented at the age of 28 y with a short history of headaches and lethargy. Investigations were compatible with a diagnosis of HUS. He has remained on haemodialysis since then and is currently well. III:8 presented at the age of 7 y with fluid retention, hypertension, and acute renal failure. He died 9 d after admission.

IV:1 presented at the age of 7 y with HUS following a viral illness. Despite treatment with peritoneal dialysis, plasma exchange, and prostacyclin, he did not recover renal function. He received a cadaver renal transplant at age 16 y, which, despite daily plasma exchange, was lost to recurrent HUS within the first two postoperative weeks. He was subsequently treated with both peritoneal dialysis and haemodialysis. He died suddenly at the age of 22 y.

Mutation screening of the genes for factor H, membrane cofactor protein, and factor I in this family had not revealed any abnormalities.

The study was approved by the Northern and Yorkshire Multi-Centre Research Ethics Committee and informed consent obtained.

### Complement Assays

Convalescent EDTA plasma samples were obtained and stored at −80 °C. C3 and C4 levels were measured by rate nephelometry (Beckman Array 360; Beckman, Fullerton, California, United States). Factor H levels were measured by radioimmunodiffusion (Binding Site, Birmingham, UK).

### *CFH* Genomic DNA Sequencing

*CFH* gDNA sequencing was undertaken as previously described [6] apart from exon 23, where the primers used were: forward 5′-CCTAATTCTCATACATTAAACATCG-3′ and reverse 5′-CAACCGTTAGTTTTCCAGGA-3′. All primers incorporated a 5′ “UNISEQ” M13-derived 17 bp tail that allowed subsequent sequencing using a common forward (5′-GTAGCGCGACGGCCAGT-3′) and reverse (5′-CAGGGCGCAGCGATGAC-3′) primer. PCR products were purified using magnetic microparticles (AMPure, Beckman) to remove unincorporated dNTPs, primers, and salts. Sequencing reactions were carried out by dye terminator cycle sequencing (DTCS kit, Beckman) using UNISEQ primers, purified using magnetic microparticles (CleanSeq, Beckman), and electrophoresed on a fluorescent 16 capillary sequencer (Beckman CEQ 8000).

The reference nucleotide sequence for *CFH* was taken from GenBank RefSeq (http://www.ncbi.nlm.nih.gov/RefSeq/) file NM_000186.1, and the nucleotide numbering uses the A of the ATG translation initiation start site as nucleotide +1. The factor H amino acid numbering includes the 18-residue signal peptide. The reference nucleotide sequence for *CFHL1* was taken from Genbank RefSeq file NM_002113.1, and the nucleotide numbering uses the A of the ATG translation initiation start site as nucleotide +1. CFH exons have been numbered 1–23 according to Rodriguez de Cordoba et al. [14]. In this nomenclature, exon 10 contributes to the transcript for factor H-like protein 1 but not factor H. The factor H-related protein 1 amino acid numbering includes the 18-residue signal peptide.

### *CFH* cDNA Sequencing

**Figure 3:**
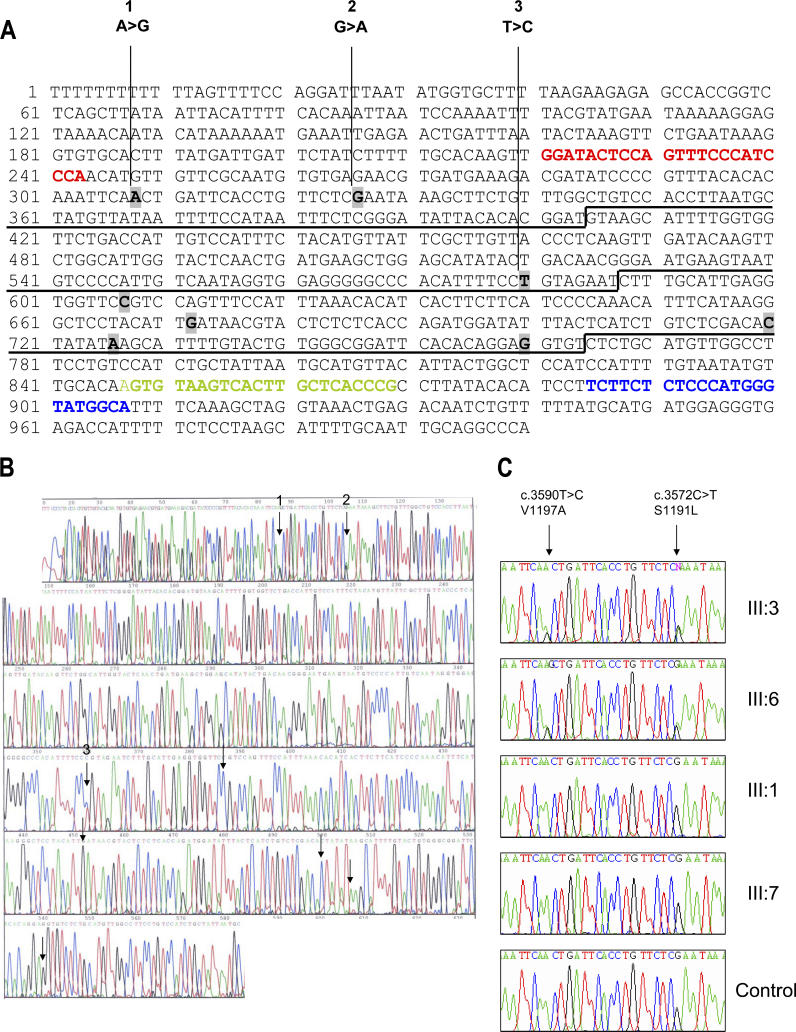
cDNA Evidence of a Hybrid Gene (A) Inverted *CFH* exons 20–23 cDNA sequence showing the site of the first-round forward primers (blue), the second round forward primers (green), and the reverse primers for both rounds (red). The nucleotides at which the *CFH* and *CFHL1* sequences differ are shown in bold and highlighted (excluding exon 20). (B) Inverted cDNA sequence of *CFH* exons 20–23 from III:6 (affected family member) showing evidence for a hybrid *CFH/CFHL1* gene. Positions at which the *CFH* and *CFHL1* sequences differ are indicated by arrows. At the three differences in exons 22 and 23 (numbered 1–3) there is a heterozygous base change, one allele being wild-type *CFH* and the other the equivalent base from *CFHL1*. (C) Inverted cDNA sequence showing hybrid *CFH/CFHL1* sequence (c.3590T>C, V1197A, and c.3572C>T, S1191L) in III:6 (affected) and III:3 (unaffected carrier) compared to normal *CFH* sequence in III:1 (unaffected), III:7 (unaffected), and a normal unrelated control.

For cDNA sequencing of *CFH* (Figure 3) mRNA was extracted from peripheral blood lymphocytes of family members (affected and unaffected) and unrelated controls. cDNA was prepared in a standard manner and *CFH* exons 21–23 amplified by seminested PCR. The forward primers were designed to be specific for *CFH* and were therefore sited in exon 20, which is not homologous to *CFHL1*. The reverse primer was complementary to both exon 23 of *CFH* and exon 6 of *CFHL1* (Figure 3A). The first-round forward primer was 5′-GCCATACCCATGGGAGAGAAGA-3′ and the second-round forward primer was 5′-CGGGTGAGCAAGTGACTTACACT-3′. The reverse primer for both rounds was 5′-GGATACTCCAGTTTCCCATCCCA-3′. Each primer incorporated a “UNISEQ” tail as before to enable sequencing with UNISEQ primers.

### Identification and Screening of the Breakpoint in *CFH/CFHL1* Hybrid Gene

**Figure 4:**
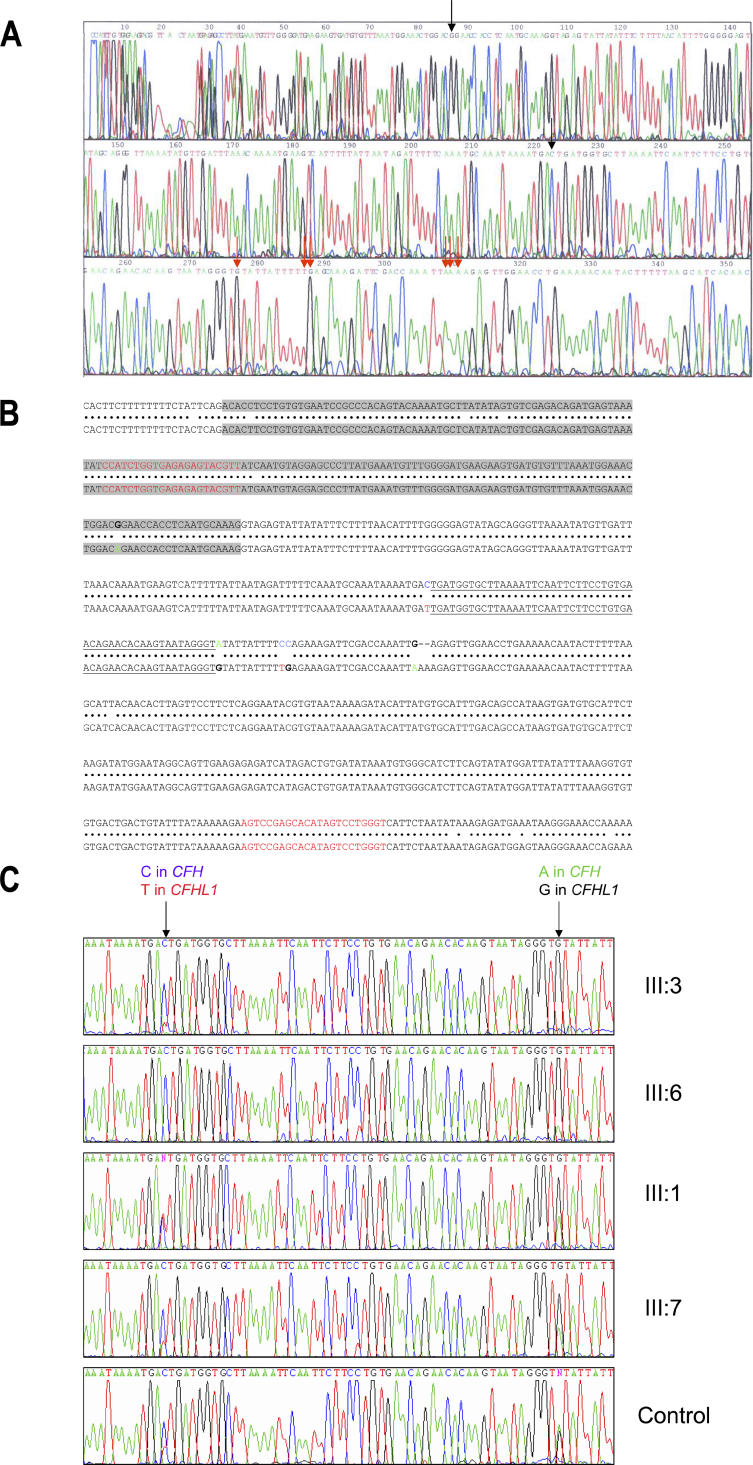
Identifying and Screening the Breakpoint Region (A) Sequence of unique PCR product generated with specific *CFH* (forward) and *CFHL1* (reverse) primers from III:6 (affected) demonstrating the hybrid product. Unique *CFH* positions are indicated with black arrows, and unique *CFHL1* nucleotides are indicated with red arrows. (B) The genomic sequence of *CFH* is shown aligned above *CFHL1*. Exons 21 and 4 of the two genes respectively are highlighted in grey. The primer-binding sites for the PCR are shown in red. The differences visible in intron 21/4 from sequencing the product are highlighted in the standard base colours A (green), C (blue), G (black), and T (red). The breakpoint is within the region underlined. (C) Sequence of the intron between exons 21 and 22 shows a switch from heterozygosity at *CFH/CFHL1* unique bases to a *CFHL1* sequence in III:3 (unaffected carrier) and III:6 (affected) compared with III:1 (unaffected), III:7 (unaffected), and a normal unrelated control.

To identify and screen the breakpoint (Figure 4), first we amplified genomic DNA across the intron between exons 21 and 22 (*CFHL1* exon 5) of the *CFH/CFHL1* hybrid gene using primers specific for *CFH* (forward) and *CFHL1* (reverse) sequence.

Next, we screened the identified breakpoint in the affected family using the primer pair 5′-CCATCTGGTGAGAGAGTACGTT-3′ (forward) and 5′-ACCCAGGACTATGTGCTCGGACT-3′ (reverse) (Figure 4B), which anneal to both *CFH* and *CFHL1*. Each primer incorporated a “UNISEQ” tail as before to enable sequencing with UNISEQ primers.

### *CFH* Dosage Analysis by Quantitative Fluorescent PCR

Two multiplex PCR assays were designed to simultaneously amplify exons 20–23 of *CFH*. PCRs were carried out in 25 μl volumes using 150 ng of DNA and contained 0.5 mM each dNTP, 6.7 mM MgCl_2_, 12.5 pmol of each primer, 1 U of a hot start Taq polymerase (Immolase, Bioline) in a buffer of 16 mM (NH_4_)_2_SO_4_, 67 mM Tris-HCl, 0.01% Tween-20. PCR cycling conditions were such that amplification remained in the linear phase of the reaction (95°C for 7 min; 20 cycles of: 94 °C for 45 s, 60 °C for 45 s, 72 °C for 1 min; and a final extension of 10 min at 72 °C). Primers that amplify *MLH1* exon 14 and *BRCA1* exon 16 were included in the assay as controls for normal dosage. One of each pair of primers was fluorescently labelled (5′FAM), and all primers are shown in Table 1. After PCR amplification products were analysed by capillary electrophoresis (ABI, Perkin Elmer). Peak areas were obtained for each sample and dosage quotients calculated.

**Table 1:**
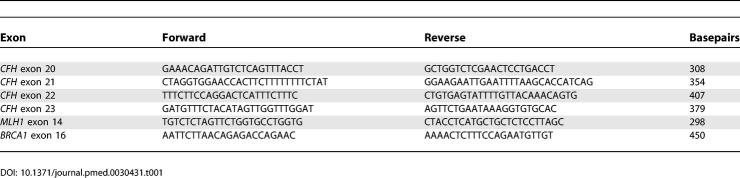
Primer Sequences for the *CFH* QF-PCR Dosage Analysis

### *CFH* Multiplex Ligation-Dependent Probe Amplification

**Figure 5:**
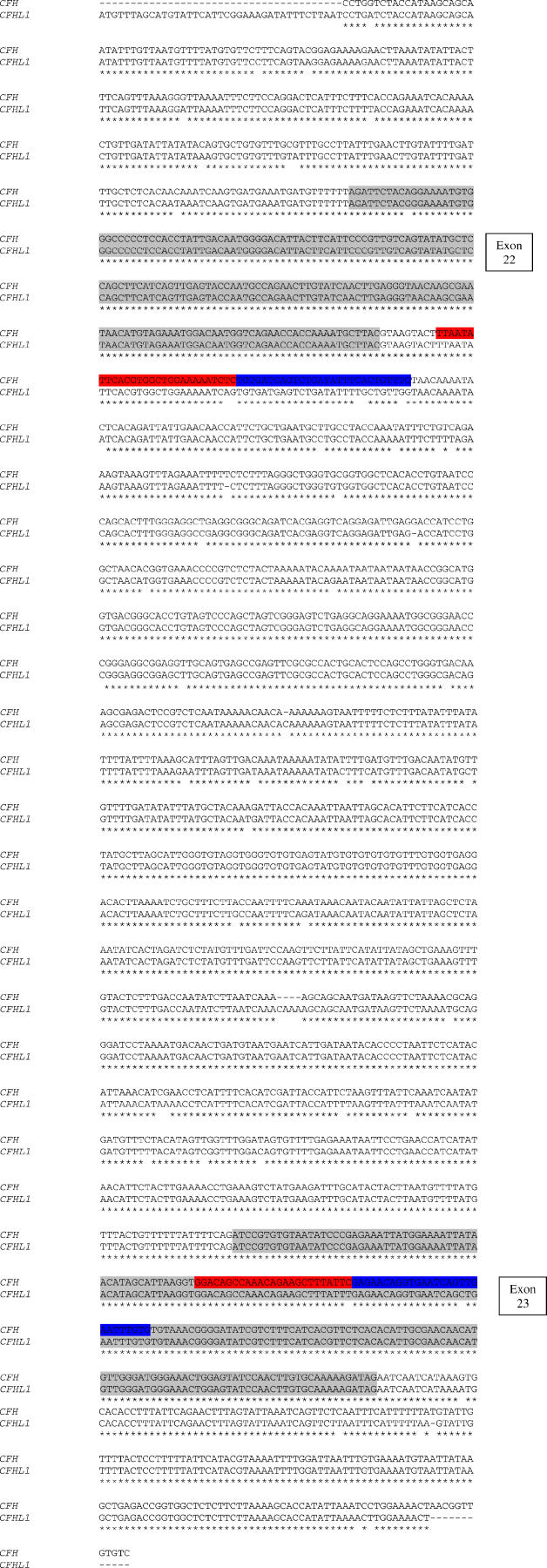
MLPA Binding Sites Used to Identify Deletions of *CFH* Exons 22 and 23 The hybridisation sequence for the 5′ and 3′ probes are shown by red and blue, respectively. The genomic sequence of *CFH* is shown aligned above *CFHL1*.

The multiplex ligation-dependent probe amplification (MLPA) reaction has been described [15]. In this study a completely synthetic probe set was used, obviating the need for a cloning step in the production of probes. Probes were designed to determine dosage for a range of *CFH* exons, along with control probes for *MSH2* exon 1 and *MLH1* exon 19 (NM_000251, NM_000249). Each probe pair hybridises to immediately adjacent targets at the sequence of interest. Hybridisation sequences are shown in Figure 5 and Table 2. Probe pairs also contained binding sites for primers used in the MLPA reaction, as well as stuffer sequence to ensure that each amplified probe product is of a unique length. Oligonucleotides were obtained from TAG Newcastle Limited (Gateshead, UK [now available at VH Bio: http://www.vhbio.com/home.aspx]). 5′ probes were RP-column purified. 3′ probes were 5′-phosphorylated and purified by desalting.

**Table 2:**
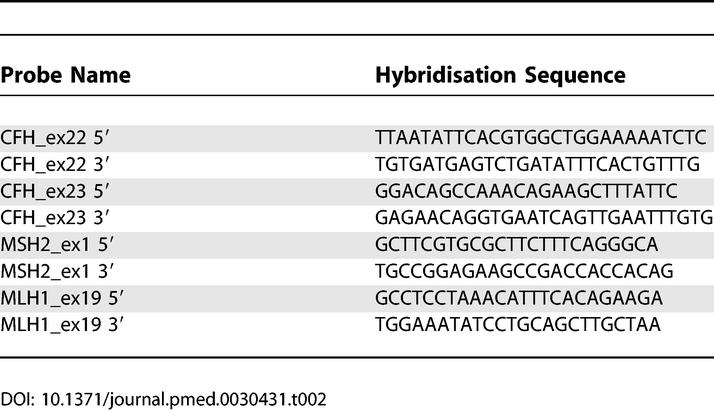
Hybridisation Sequences for the *CFH* Exons 22/23 MLPA Probes

Reagents for the MLPA reaction were purchased from MRC-Holland (Amsterdam, the Netherlands). The ligation reactions were carried out according to the manufacturer's recommended protocol using 100–200 ng of genomic DNA and 2 fmol of probe. Incubations and PCR amplifications were carried out on a DNA Engine Tetrad 2 thermal cycler (BioRad Laboratories, Hercules, California, United States). Amplified products were diluted 1 in 10 to give peak heights within the quantitative range (approximately 100–4,000 units) on the ABI PRISM 3130 Genetic Analyzer capillary electrophoresis system (Applied Biosystems, Foster City, California, United States). For size standards, 1 μl of diluted product and 0.5 μl of ROX 500 internal size standard were made up to 10 μl using dH_2_O, and samples were run on the ABI 3130. Peak areas for each sample were determined using the proprietary Genemapper software and dosage quotients calculated.

### Southern Blots

**Figure 6:**
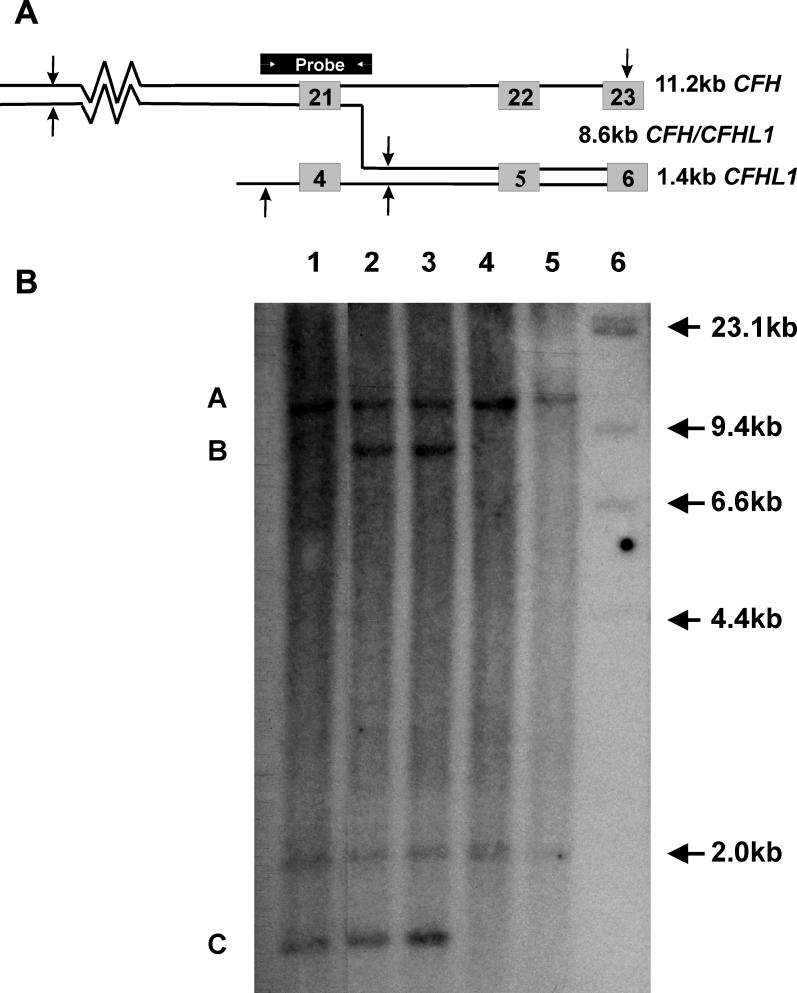
Southern Blot Evidence of Genomic Rearrangement (A) A Southern blot using a 1.1 kb probe overlying *CFH* exon 21 and *CFHL1* exon 4 hybridised to HindIII-digested DNA (sites shown as arrows) will result in fragments of 11.2 kb from *CFH,* 1.4 kb from *CFHL1,* and 8.6 kb from a *CFH/CFHL1* hybrid gene. The site of the 1.1 kb probe is indicated above. (B) Southern blot showing an additional 8.6 kb band (indicated by B) in lanes 2 and 3, which represent III:3 (unaffected carrier) and III:6 (affected) compared to lanes 1, 4, and 5, which represent III:1 (unaffected) and individuals with homozygous deletion of *CFHL1*. Bands at A and C represent fragments of 11.2 kb from *CFH* and 1.4 kb from *CFHL1,* respectively. A size ladder is shown to the right with heavy arrows indicating the expected sizes.

To provide further confirmation that the point of recombination responsible for the *CFH/CFHL1* hybrid gene lies within the intron between *CFH* exons 21/22 and *CFHL1* exons 4/5, Southern blots were done using a 1.1 kb probe (Figure 6). The putative crossover leading to the formation of the *CFH/CFHL1* hybrid gene is shown as a line that crosses over 110–150 bases into the introns between exons 21/22 of *CFH* and 4/5 of *CFHL1*. A DNA probe was prepared by PCR amplification of a 1.1 kb region spanning the putative breakpoint using the following primers: forward, 5′-GTAGCGCGACGGCCAGTGGTCTATCAGTGTTCTAGCGAAGGA-3′ and reverse, 5′-GTGCACCAGTTAACAGGCCAT-3′.

Genomic DNA (10 μg) from two affected individuals, one unaffected individual, and two individuals who were homozygously deleted for *CFHL1* was digested with HindIII. Electrophoresis was carried out overnight in a 0.8% agarose gel in 1× TAE buffer (0.04 M Tris-acetate buffer, 0.001 M EDTA) at 40 mA. DNA was transferred to a nylon membrane (Hybond N+, Amersham [http://www.amersham.com/]) after denaturation in 1.5 M NaCl, 0.5 M NaOH and neutralisation in 0.4 M Tris base, 0.25 M trisodium citrate, 2.5 M NaCl. The DNA probe (20 ng) was labelled using the Rediprime system (Amersham). Hybridisations were carried out overnight at 65 °C in a mix containing 5× SSC (0.75 M NaCl, 0.075 M trisodium citrate), 5× Denhardt's (0.1% PVP, 0.1% BSA, and 0.1% Ficoll), 0.1% SDS, 0.1% sodium pyrophosphate, 10% dextran sulfate, and 100 μg/ml sonicated denatured salmon sperm DNA. The filter was washed at 65 °C to a final stringency of 0.5× SSC (including 0.1% SDS) and autoradiographed with intensifying screens for 12 h.

## Results

### Complement Profiles

Serum levels of C3, C4, and factor H are shown in Table 3. Factor H levels were normal in all family members, as was expected, because the secreted product of the hybrid gene is identical to the factor H mutant c.3572C>T, S1191L/c.3590T>C, V1197A.

**Table 3:**
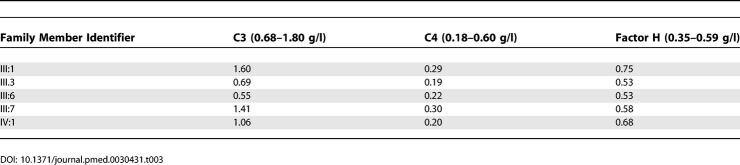
Complement Levels

### *CFH* gDNA Sequencing

Sequencing of genomic *CFH* DNA from II:8 (unaffected carrier), III:1 (unaffected), III:3 (unaffected carrier), III:6 (affected), III:7 (unaffected) and IV:1 (affected) showed wild-type sequence. This was expected, because the hybrid *CFH/CFHL1* gene described here would not be picked up by genomic sequencing using *CFH*-specific primers

### *CFH* cDNA Sequencing

In III:3 (unaffected carrier) and III:6 (affected), cDNA sequencing showed two heterozygous changes c.3572C>T and c.3590T>C in exon 23, leading to S1191L and V1197A, respectively (Figure 3B and 3C). In addition, there was a heterozygous synonymous change in exon 22. These changes were not found in III:1, III:7 (both unaffected), or an unrelated control.

### Identification and Screening of the Breakpoint in the *CFH/CFHL1* Hybrid Gene

The DNA breakpoint in III:6 (affected) was identified by sequencing PCR products using specific *CFH* (forward) and *CFHL1* (reverse) primers spanning the intron between exons 21 and 22 (*CFHL1* exon 5) of the *CFH/CFHL1* hybrid gene. This generated a unique product, sequencing of which showed the breakpoint to be in a 52 bp section starting 118 bp into intron 21. The 52 bp section is defined by differences between *CFH* and *CFHL1* sequence (Figure 4A). Screening of other family members using primers spanning the breakpoint and designed to anneal to both *CFH* and *CFHL1* (Figure 4B) confirmed the breakpoint. Sequence of this region (Figure 4C) shows a switch from heterozygosity at *CFH/CFHL1* unique bases to *CFHL1* sequence in III:3 (unaffected carrier) and III:6 (affected) compared with III:1 (unaffected), III:7 (unaffected), and a normal unrelated control.

### *CFH* Dosage Analysis by Quantitative Fluorescent PCR and MLPA

**Figure 7:**
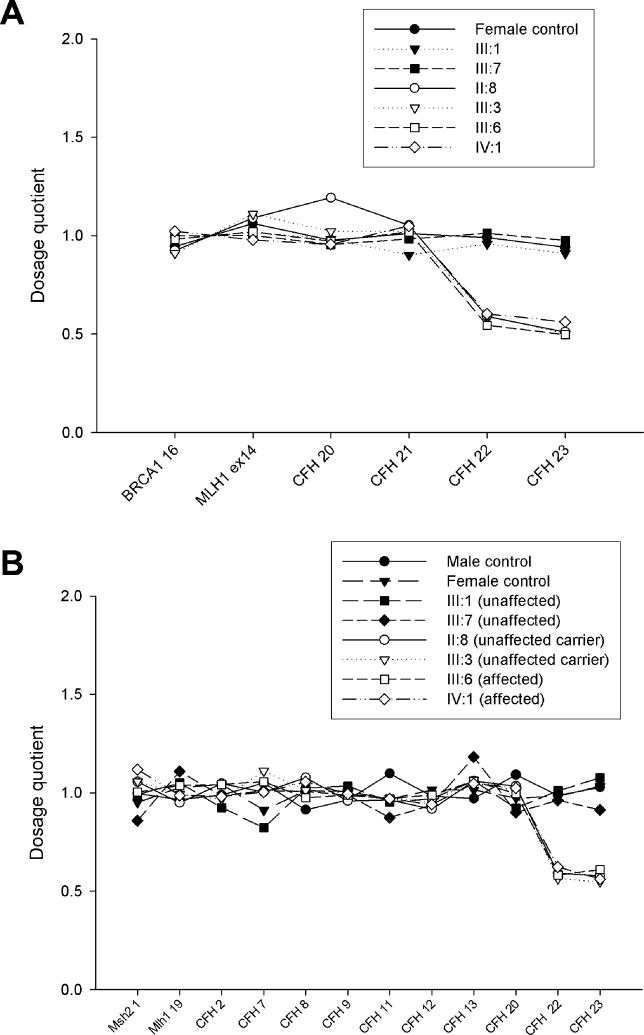
Dosage Evidence of a Hybrid Gene (A) QF-PCR and (B) MLPA dosage quotients showed an apparent heterozygous “deletion” of *CFH* exons 22 and 23 in II:8 (unaffected carrier), III:3 (unaffected carrier), III:6 (affected), and IV:1 (affected). There was no evidence of a “deletion” in III:1 and III:7 (both unaffected).

*CFH* dosage quotients showed an apparent heterozygous “deletion” of *CFH* exons 22 and 23 in II:8 (unaffected carrier), III:3 (unaffected carrier), III:6 (affected), and IV:1 (affected). There was no evidence of a “deletion” in III:1 and III:7 (both unaffected) (Figure 7).

In a panel of 80 patients with aHUS, three further individuals were identified by MLPA as having a deletion of *CFH* exons 22 and 23. In all three, factor H levels were normal and *CFH* gDNA sequencing had shown normal wild-type sequence, but presence of a hybrid *CFH/CFHL1* gene was confirmed by *CFH* cDNA sequencing. This showed the same changes identified in III:3 and III:6.

In a panel of 100 normal control individuals, MLPA showed no evidence of a deletion of *CFH* exons 22 and 23.

### Southern Blot

In III:3 (unaffected carrier) and III:6 (affected) a Southern blot with HindIII showing an additional 8.6 kb band (the band at “B” in Figure 6B). This additional band is derived from the hybrid *CFH/CFHL1* gene as shown in Figure 6A.

## Discussion

In this study we have provided conclusive evidence that LCR nonhomologous recombination in the RCA cluster of genes has resulted in the formation of a hybrid *CFH/CFHL1* gene that predisposes to the development of aHUS. The protein product of the hybrid gene is identical to the factor H mutant c.3572C>T, S1191L/c.3590T>C, V1197A which we have shown arises by gene conversion in aHUS [10]. We have previously shown that this mutant is functionally significant, in that binding to C3b is impaired. The hypothesis for the existence of a hybrid *CFH/CFHL1* gene in the family described in this manuscript was based on two observations. First, we knew from our original linkage study [1] that the affected individuals in this family mapped to the RCA cluster at position 1q32. Moreover, in this family there was a strong history of disease recurrence following kidney transplantation, suggesting that a soluble circulating complement regulator was responsible. Second, our recent observation that LCRs in the RCA cluster predisposed to gene conversion events suggested that LCR nonhomologous recombination might also be occurring. In support of this was the observation that the presence of factor H-related protein 1 (encoded by *CFHL1*) in the serum of normal controls is polymorphic, with 4.4% of healthy blood donors having complete deficiency [12]. We hypothesised that the crossover responsible for deletion of *CFHL1* could also potentially result in formation of a *CFH/CFHL1* hybrid gene. The evidence presented here strongly supports this hypothesis. Moreover, we have evidence from three other unrelated aHUS patients that the phenomenon is not unique to this family. Renal transplantation in patients with a *CFH* mutation is associated with an 80% risk of the graft being lost to recurrent disease within two years of transplantation [16]. It is currently recommended that all patients undergo *CFH* genotyping prior to transplantation. However, the hybrid *CFH/CFHL1* gene described here would not be picked up by genomic sequencing using *CFH*-specific primers. We would, therefore, now recommend that all patients be screened also with *CFH* MLPA.

Could the results of this study have implications for other complement-related diseases? It has been established in several independent cohorts that *CFH* alleles act as a susceptibility factor for age-related macular degeneration [17–20] and type II membranoproliferative glomerulonephritis [21]. It would be fascinating to test the hypothesis that copy number of *CFHL1* and *CFHL3* acts as a susceptibility factor for such diseases. It is tempting to speculate that such an effect might be mediated by an interaction between factor H and the factor H-related proteins.

## Supporting Information

### Accession Numbers

Online Mendelian Inheritance in Man (http://www.ncbi.nlm.nih.gov/entrez/query.fcgi?db=OMIM) accession numbers for the genes and conditions discussed in this article are aHUS (235400), *CFH* (134370), *CFHL1* (134371), *CFHL2* (600889), *CFHL3* (605336), *CFHL4* (OMIM 605337), and *CFHL5* (OMIM 608593). The GenBank (http://www.ncbi.nlm.nih.gov/) accession numbers for other genes are *MSH2* exon 1 (NM_000251) and *MLH1* exon 19 (NM_000249).

## Abbreviations

aHUS: atypical haemolytic uraemic syndrome
LCR: low copy repeat
MLPA: multiplex ligation-dependent probe amplification
RCA: regulator of complement activation
SCR: short consensus repeat
